# Use of transcranial magnetic stimulation to assess relaxation rates in unfatigued and fatigued knee-extensor muscles

**DOI:** 10.1007/s00221-020-05921-9

**Published:** 2020-11-02

**Authors:** Gianluca Vernillo, Arash Khassetarash, Guillaume Y. Millet, John Temesi

**Affiliations:** 1 Human Performance Laboratory, Faculty of Kinesiology, University of Calgary, Calgary, AB Canada; 2 Department of Biomedical Sciences for Health, Università Degli Studi di Milano, Milan, Italy; 3 University of Lyon, UJM Saint-Etienne, Inter-University Laboratory of Human Movement Biology, EA 7424), 42023 Saint-Etienne, France; 4 Institut Universitaire de France (IUF), Paris, France; 5 Faculty of Health and Life Sciences, Northumbria University, Newcastle upon Tyne, UK

**Keywords:** Fatigue, Knee extensors, Transcranial magnetic stimulation, Muscle relaxation rate

## Abstract

We examined whether transcranial magnetic stimulation (TMS) delivered to the motor cortex allows assessment of muscle relaxation rates in unfatigued and fatigued knee extensors (KE). We assessed the ability of this technique to measure time course of fatigue-induced changes in muscle relaxation rate and compared relaxation rate from resting twitches evoked by femoral nerve stimulation. Twelve healthy men performed maximal voluntary isometric contractions (MVC) twice before (PRE) and once at the end of a 2-min KE MVC and five more times within 8 min during recovery. Relative (intraclass correlation coefficient; ICC_2,1_) and absolute (repeatability coefficient) reliability and variability (coefficient of variation) were assessed. Time course of fatigue-induced changes in muscle relaxation rate was tested with generalized estimating equations. In unfatigued KE, peak relaxation rate coefficient of variation and repeatability coefficient were similar for both techniques. Mean (95% CI) ICC_2,1_ for peak relaxation rates were 0.933 (0.724–0.982) and 0.889 (0.603–0.968) for TMS and femoral nerve stimulation, respectively. TMS-induced normalized muscle relaxation rate was − 11.5 ± 2.5 s^−1^ at PRE, decreased to − 6.9 ± 1.2 s^−1^ (− 37 ± 17%, *P* < 0.001), and recovered by 2 min post-exercise. Normalized peak relaxation rate for resting twitch did not show a fatigue-induced change. During fatiguing KE exercise, the change in muscle relaxation rate as determined by the two techniques was different. TMS provides reliable values of muscle relaxation rates. Furthermore, it is sufficiently sensitive and more appropriate than the resting twitch evoked by femoral nerve stimulation to reveal fatigue-induced changes in KE.

## Introduction

Muscle relaxation is an important component of movement control, particularly during movements in which muscle activation has to switch between different contracting muscles (Buccolieri et al. 2004). Muscle relaxation depends on the rate of detachment of cross-bridges during the relaxation process (Houston et al. 1987) and represents the sum of all processes at the level of the skeletal muscle that follow the cessation of the neural drive to the muscle fibres, providing information about the intrinsic properties of muscle fibres (Dux 1993). However, to date the scientific literature has emphasised muscle contraction, while muscle relaxation is often overlooked (Kortman et al. 2012).

In humans, the properties of muscle fibres are commonly assessed by measuring the resting twitch evoked by a supramaximal electrical stimulus of the peripheral nerve or intramuscular nerve fibres in the relaxed muscle state (Millet et al. 2011). The characteristics of the resting twitch provide information about both the speed of muscle contraction and relaxation. Further, these characteristics provide insight into the force output from the muscle (Todd et al. 2007). However, the relevance of this technique has been questioned since it only reveals properties of the muscle at rest while muscle properties are most functionally relevant during a voluntary contraction, when the central nervous system is actively driving the muscle (Todd et al. 2007).

To overcome this issue, transcranial magnetic stimulation (TMS) delivered to the motor cortex may offer a valuable alternative. TMS is a non-invasive technique that can be used to excite or inhibit different cortical areas of the human brain. When single-pulse TMS of sufficient intensity is delivered to the motor cortex during a voluntary contraction, it induces transient excitation in both the electromyography (EMG) (i.e., motor-evoked potential) and mechanical (force) responses (i.e., superimposed twitch) of the target muscle. Following the motor-evoked potential, there is a period of near-silence in the EMG termed the silent period. As a result of the withdrawal of voluntary drive, muscle fibres that are voluntarily contracting relax and force decreases. Accordingly, it has been proposed to analyze the rate of muscle relaxation during the silent period elicited by TMS delivered to the motor cortex (Todd et al. 2005). This method has been applied to the finger flexors (Molenaar et al. 2018), elbow flexors (Todd et al. 2005, 2007; Hunter et al. 2006, 2008; Molenaar et al. 2013), plantar flexors (McNeil et al. 2013; Yacyshyn et al. 2017), and dorsiflexors (McNeil et al. 2013), either in an unfatigued or fatigued state. Results have shown that TMS can be used to measure relaxation rates in the above-mentioned muscle groups.

However, a direct comparison between TMS-induced muscle relaxation rate and the relaxation rate determined from the resting twitch evoked by femoral nerve stimulation has not been reported for the knee extensors (KE). It is possible that TMS-induced muscle relaxation rate behaves differently for KE, when compared with other muscles, due to different somatotopic organization and recruitment thresholds (Leung et al. 2018; Krishnan 2019), functional role (Maffiuletti et al. 2008) as well as neuromuscular aspects (Brouwer and Ashby 1990; Saltin and Gollnick 2011; Vernillo et al. 2018; Temesi et al. 2019). Therefore, understanding whether TMS is a valid technique that can be used for measuring KE relaxation rate is important because KE is (1) responsible for knee-extensor force production and therefore plays a key role during ambulatory, functional and sport activities (Maffiuletti et al. 2008); and (2) commonly used in studies investigating muscle fatigue with TMS (e.g., Sidhu et al. 2009; Goodall et al. 2012; Klass et al. 2012; Temesi et al. 2013; Vernillo et al. 2018). Furthermore, the use of TMS, as opposed to peripheral electrical stimulation, to assess muscle relaxation rate in KE would allow muscle contractile properties to be examined while receiving drive from the central nervous system (Todd et al. 2007).

Therefore, the aim of this study was to assess whether TMS is appropriate for measuring muscle relaxation rate in KE. An important characteristic of any measurement must be close agreement between consecutive measurements in one participant (repeatability) and small measurement error compared with the true difference between participants (reliability) (Bartlett and Frost 2008). Accordingly, we compared the repeatability and reliability of peak muscle relaxation rates calculated from the falling phase of the resting twitch evoked by femoral nerve stimulation and the decrease in force during the period of EMG silence after delivery of TMS during KE maximal voluntary contractions in healthy participants. Furthermore, in response to a sustained KE maximal voluntary contraction, we assessed the ability of TMS to measure the time course of changes in the muscle relaxation rate with the development of fatigue.

## Methods

### Participants

Twelve healthy and physically active males (age 31 ± 9 years; height 179 ± 7 cm; body mass 75 ± 9 kg) volunteered for this study. Exclusion criteria for participation were injury to the lower limbs during the previous 6 months, history of heart disease or hypertension, and contraindications to TMS (Rossi et al. 2011). Participants were instructed to avoid the consumption of caffeine on the day of the experiment and avoid performing any strenuous exercise during the 48 h prior to testing. This study conformed to the standards set by the Declaration of Helsinki, except for registration in a database. The experimental protocol was approved by the University of Calgary Conjoint Health Research Ethics Board (#REB14-1625). Participants were informed of the experimental protocol and all associated risks prior to giving written informed consent.

### Experimental protocol

Results from some of the data collected from this protocol have previously been published (Vernillo et al. 2018, 2019, 2020; Temesi et al. 2019). Each participant completed one familiarization session and one experimental session. During the familiarization session, participants performed maximal and submaximal voluntary isometric contractions of KE with and without TMS or femoral nerve stimulation. The experimental session consisted of a 2-min sustained KE MVC. Before each 2-min MVC (PRE), two neuromuscular evaluations (separated by 60 s) with TMS and femoral nerve stimulation (see “Neuromuscular evaluation” section) were performed. Peak force from the second MVC of the neuromuscular evaluation was always within 5% of peak force from the first MVC of the neuromuscular evaluation for all participants. Mean values from the two PRE neuromuscular evaluations were used for subsequent analyses. At the end of the 2-min MVC, a neuromuscular evaluation was performed as an extension of the 2-min MVC (i.e., the participant was not permitted to relax) (POSTimm). Additional evaluations were performed 5 s after relaxation (POSTrelax), as well as 1 (POST 1), 2 (POST 2), 4 (POST 4), and 8 (POST 8) min after the end of the 2-min MVC. The two sessions were separated by between 3 and 7 days and each participant performed both sessions at the same time of day to control for within-participant diurnal variation.

#### Force recordings

All measurements were taken from the participants’ right leg. Force was measured by a calibrated force transducer (LC101-2K; Omegadyne, Sunbury, OH) with amplifier attached to the right leg by a noncompliant strap immediately proximal to the malleoli of the ankle joint. Participants were seated in a custom-built isometric ergometer in an upright position with both right knee and hips at 90° of flexion and secured by chest and hip straps. The force transducer was fixed to the chair such that force was measured in direct line to the applied force. The force was displayed on a computer screen and participants received real-time visual feedback during all voluntary contractions.

Because muscle relaxation was determined from the decrease in KE force during the silent period, the duration of the silent period was verified to ensure it was sufficient to allow for measurement of peak relaxation rates during maximal contractions. Therefore, EMG of the right *vastus lateralis*, and *rectus femoris* was recorded with pairs of self-adhesive surface electrodes (10-mm recording diameter; Meditrace 100; Covidien, Mansfield, MA) in bipolar configuration with 30-mm interelectrode distance and reference on the *patella*. Placement of EMG electrodes for *vastus lateralis* was on the distal portion of the muscle belly between the apex of the greater trochanter and the superolateral border of the *patella* and for *rectus femoris* on the distal portion of the muscle belly between the anterior superior iliac spine and the superior border of the *patella* (Botter et al. 2011). The skin where electrodes were placed was shaved, lightly abraded, and cleaned with isopropyl alcohol to achieve a low impedance level (< 5 kΩ). Force and EMG signals were analog-to-digitally converted at a sampling rate of 2000 Hz by PowerLab system (16/35, ADInstruments, Bella Vista, Australia) and octal bioamplifier (ML138; ADInstruments; common mode rejection ratio = 85 dB, gain = 500) with band pass filter (5–500 Hz) and analyzed offline using Labchart 8 software (ADInstruments).

#### Transcranial magnetic stimulation

The motor cortex was stimulated by a magnetic stimulator (Magstim 200^2^; The Magstim Company Ltd, Whitland, UK) with a 110-mm double-cone coil (maximum output of 1.4 T). Single stimuli were delivered to the contralateral motor cortex, producing an induced postero-anterior current. Every centimetre was demarcated from the vertex to 2 cm posterior to the vertex along the nasal-inion line and 1 cm laterally over the left motor cortex. Optimal coil position was determined by assessing MEP responses evoked during brief isometric voluntary contractions at 20% MVC and 50% maximal stimulator output. The optimal coil position was where the largest motor-evoked potentials in the *rectus femoris* were elicited. Optimal coil position for the session was marked on a lycra swim cap. Stimulus intensity was determined by stimulus–response curve from responses during brief isometric contractions at 20% MVC. Four consecutive contractions were performed at 15-s intervals at each of the following randomly ordered stimulus intensities: 20, 30, 40, 50, 60, 70, and 80% maximal stimulator output. Optimal stimulus intensity was defined as the lowest intensity eliciting maximal MEP amplitudes with minimal antagonist responses (Temesi et al. 2014). Mean stimulus intensity was 63 ± 9% of maximal stimulator output.

#### Femoral nerve stimulation

Resting muscle twitches were evoked by electrical stimulation (DS7A; Digitimer, Welwyn Garden City, Hertfordshire, UK). Single pulses (1-ms duration) were delivered to the femoral nerve trunk via a surface cathode taped into the femoral triangle (Meditrace 100) and a 50 × 90 mm rectangular anode (Durastick Plus; DJO Global, Vista, CA) in the gluteal fold. During femoral nerve stimulation, a small gauze ball was placed over the cathode before securing it with tape to apply pressure over the stimulation site. Stimuli were delivered incrementally in the relaxed muscle state until M-wave and twitch amplitudes plateaued. A stimulus intensity of 130% of the intensity to elicit maximal M-wave and twitch amplitudes was used throughout the experiment. The supramaximal stimulus intensity was 84 ± 36 mA.

#### Neuromuscular evaluation

The neuromuscular evaluation was previously published (Vernillo et al. 2018) and consisted of a sustained contraction comprised of an MVC followed by 75% and 50% MVC for the determination of the voluntary activation [i.e., the level of voluntary drive to the muscle (Gandevia et al. 1995)]. TMS was delivered at each force level and participants were instructed to recontract as quickly as possible to the pre-stimulus voluntary force (Mathis et al. 1998). Each sustained contraction lasted approximately 9 s (~ 3 s per contraction intensity). Immediately after the neuromuscular evaluation, a single femoral nerve electrical stimulation was delivered when the muscle was relaxed. Visual feedback of the force produced, and target force levels were provided to the participants by means of a real-time display on a computer screen. For the purpose of the present study, only the evoked twitch and TMS parameters during the 100% MVC were taken into consideration.

### Data analysis

The force traces were low-pass filtered using a 4th order Butterworth filter with zero time-lag and cut-off frequency of 10 Hz. This filtering process was necessary to remove the noise of the instantaneous slope (force derivative). The duration of *vastus lateralis* and *rectus femoris* silent periods were measured by visually inspecting the interval from the TMS stimulus to the return of continuous voluntary EMG (Taylor et al. 1996).

Responses evoked by femoral nerve stimulation in the relaxed muscle in a potentiated state were analysed for (1) amplitude of the potentiated peak twitch, (2) time to peak amplitude of the potentiated peak twitch (i.e., interval from the onset of the twitch to the peak amplitude), and (3) half-relaxation time of the potentiated peak twitch (i.e., interval between the peak amplitude and the point at which force was reduced by 50%).

**Fig. 1:**
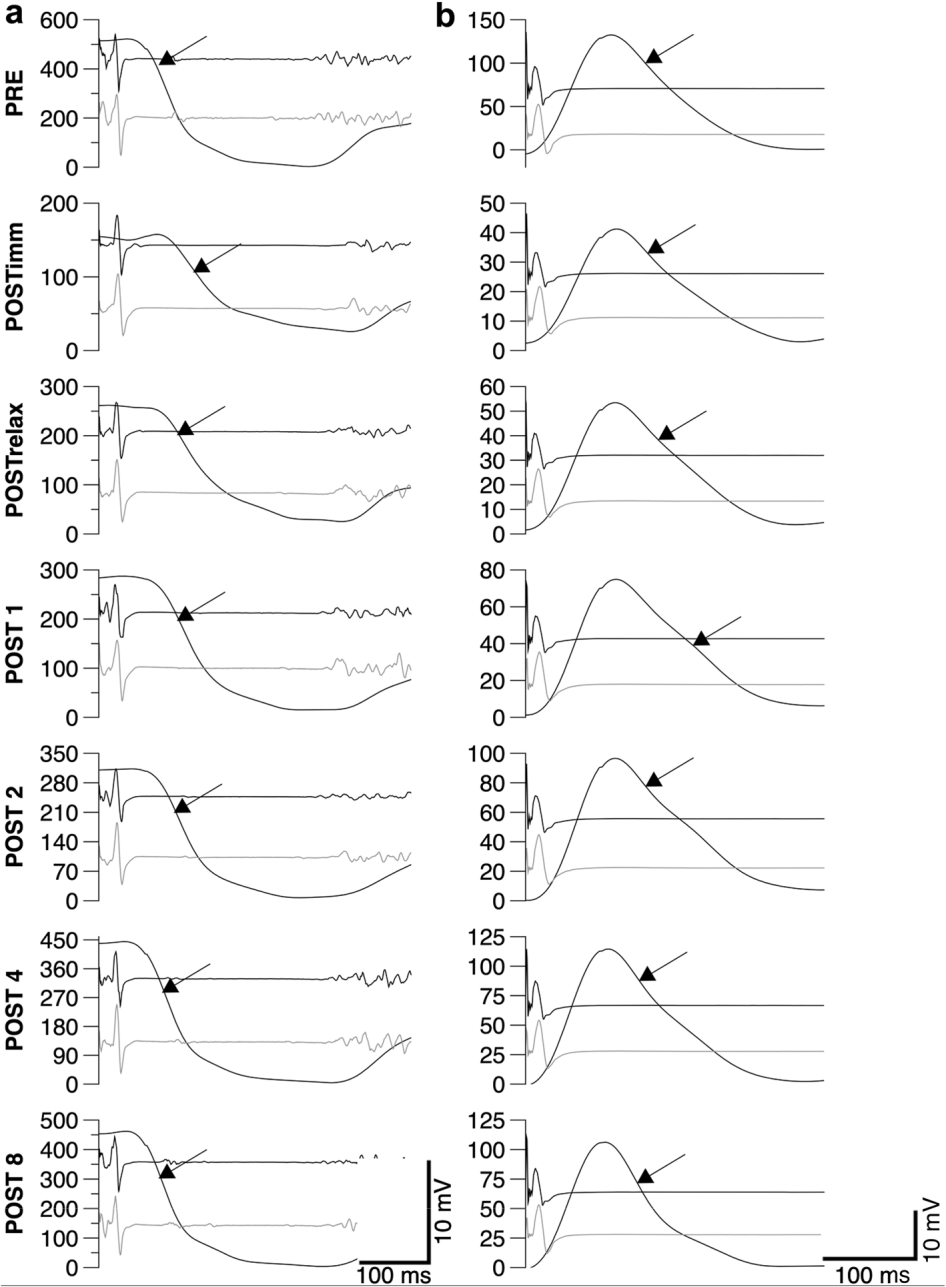
Peak muscle relaxation rates before the 2-min maximal MVC (PRE) and at the end of the 2-min MVC. After the sustained contraction, a neuromuscular function evaluation was performed as an extension of the 2-min MVC (POSTimm) and additional evaluations were performed after 5 s of relaxation (POSTrelax) and 1 (POST 1), 2 (POST 2), 4 (POST 4), and 8 (POST 8) min after the end of the 2-min MVC. Peak muscle relaxation rates were calculated from the decrease in force during the silent period during maximal voluntary contractions (**a**), and from the falling phase of the resting twitch evoked by femoral nerve stimulation (**b**). Stimuli were delivered at time 0 ms. Peak rate of relaxation was calculated as the negative slope over a 10-ms interval (5 ms either side of the steepest instantaneous slope). To account for differences in both voluntary strength and evoked twitch amplitude, normalized rates of relaxation were calculated by dividing the absolute rates of relaxation by the peak force which preceded the relaxation. EMG traces for *rectus femoris* (black traces) and *vastus lateralis* (grey traces) show muscular responses evoked by TMS (**a**) and femoral nerve stimulation (**b**). Force and EMG traces are from a single participant (33-year-old man). Arrows indicate the time at which the peak relaxation rate occurred. Different scales have been used for *y*-axes for illustrative purposes

Muscle relaxation rates were calculated from the decrease in force during the silent period following TMS delivery or the falling phase of the resting twitch evoked by femoral nerve stimulation (Fig. 1). In all instances, the peak rate of muscle relaxation was calculated as the negative slope over a 10-ms interval (5 ms either side of the steepest instantaneous slope) (e.g., Todd et al. 2005, 2007; McNeil et al. 2013). To account for differences in both voluntary strength and evoked twitch amplitude within and between participants, normalized rates of relaxation were calculated by dividing the absolute rates of relaxation by the peak force which preceded the relaxation. This value reflects the relative peak relaxation rate of all knee-extensor muscles that contribute to the measured force (voluntary plus evoked) and that are suppressed by the inhibitory effects of TMS (Todd et al. 2005, 2007; Hunter et al. 2006, 2008; McNeil et al. 2013; Yacyshyn et al. 2017). Furthermore, time to peak relaxation was assessed as the time from TMS stimulus until the moment of peak relaxation (Molenaar et al. 2013).

### Statistical analysis

Absolute reliability is the variability due to random error (Ludbrook 2002) and is consequently influenced by the degree to which measurements vary (with the assumption that with lower variability, reliability is higher) (Vaz et al. 2013). To quantify absolute reliability in the measurement error in unfatigued KE, the repeatability coefficient (RC, also referred to as the smallest real difference) was determined. RC is the value below which the absolute differences between two subsequent measurements would lie with 95% probability (Beckerman et al. 2001; Vaz et al. 2013) and was calculated as:

$${\text{RC}} = 2.77 \times {\text{S}}_{\text{w}},$$

where ${\text{S}}_{\text{w}}$ is the within-participant standard deviation and 2.77 is obtained by multiplying √2 times 1.96 (Beckerman et al. 2001; Vaz et al. 2013). Furthermore, within-participant variability was assessed by calculating the coefficient of variation (CV), defined as the ratio of the within-participant standard deviation of the mean (Atkinson and Nevill 1998). CV for all participants was calculated for all variables of interest as the within-participant standard deviation divided by mean of the two measurements. The mean of all CV was considered as the overall within-participant coefficient of variation. To compare both absolute and within-participant reliability in unfatigued KE, paired *t* tests were performed between peak muscle relaxation rates determined via responses elicited by TMS and femoral nerve stimulation. Two-way random effects, absolute agreement intra-class correlation coefficients (ICC_2,1_) were also calculated to determine relative reliability, defined as the size of the within-participant measurement error to the inherent between-participants variability (Atkinson and Nevill 1998; Vaz et al. 2013). ICC_2,1_ are classified as poor (< 0.50), moderate (0.50–0.75), good (0.75–0.90) and excellent (> 0.90) (Koo and Li 2016).

To test differences between PRE and POSTimm, as well as during the recovery time, a longitudinal analysis was performed using generalized estimating equations (GEE; i.e., GEE under ‘generalized linear model’ procedure in SPSS v. 26) to take into account the correlated nature of observations within each participant (i.e., within-participant measurements) (Liang and Zeger 1986). If a significant main effect for time was observed, Bonferroni’s test was used for post hoc analysis. Statistical analyses were conducted using IBM™ SPSS™ Statistics (version 26.0.0; IBM Corp., Somers, New York, NY) with the criterion *α* level set to 0.05.

## Results

### Repeatability and reliability in unfatigued knee-extensor muscles

**Fig. 2:**
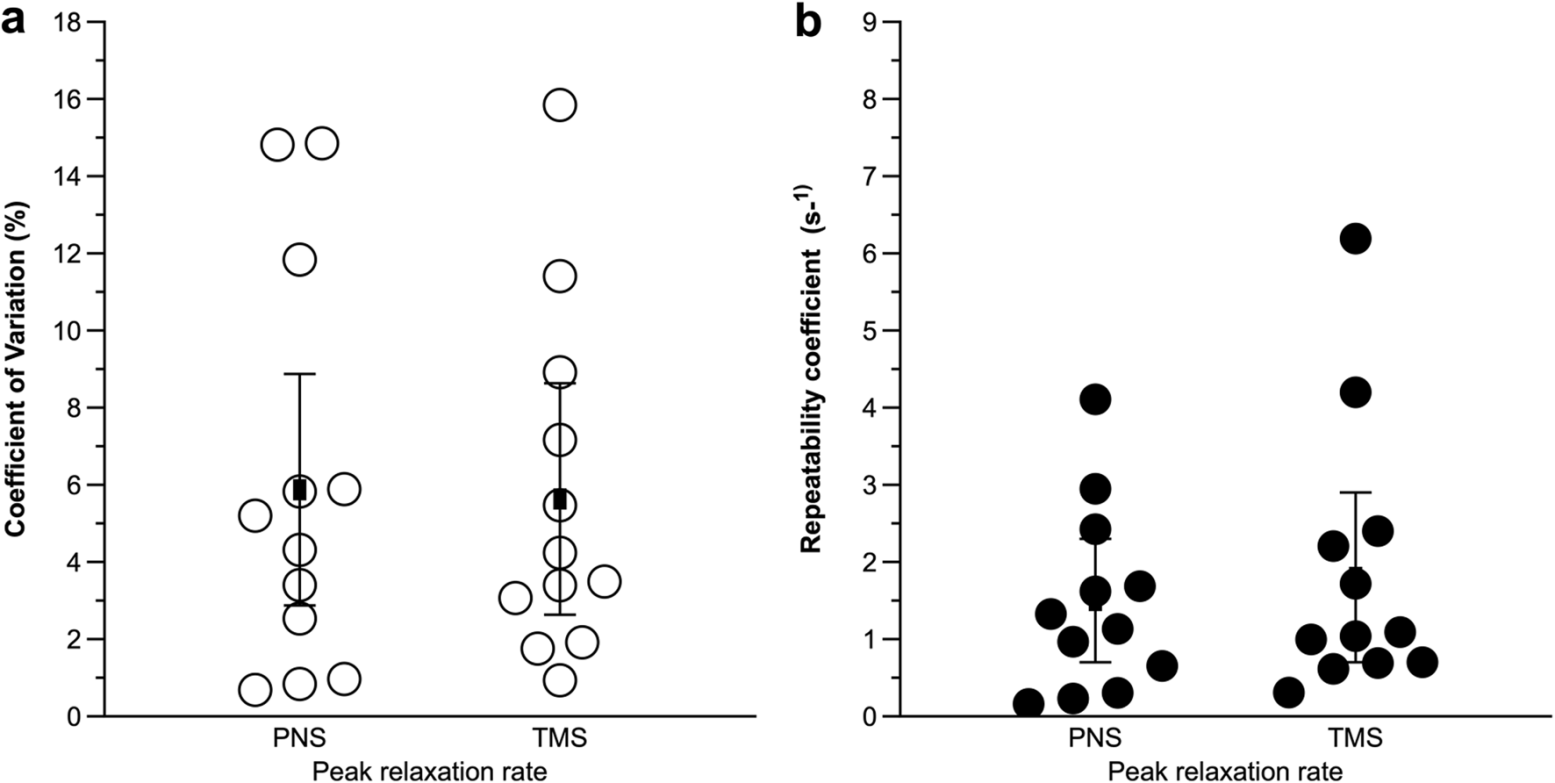
Comparison of coefficient of variation (**a**), and repeatability coefficient (**b**) of peak muscle relaxation rates determined from the falling phase of the resting twitch evoked by femoral nerve stimulation (PNS), and the decrease in force during the silent period during maximal voluntary contractions (TMS). Circles represent individual data, black squares means, and error bars 95% confidence intervals. Different scales have been used for *y*-axes for illustrative purposes

All relaxation properties showed similar CV and RC whether elicited by TMS or femoral nerve stimulation (Table 1 and Fig. 2). Furthermore, mean (95% CI) ICC_2,1_ for peak relaxation rates were 0.933 (0.724–0.982, rated moderate to excellent) for TMS-induced relaxation and 0.889 (0.603–0.968, rated moderate to excellent) for resting twitches evoked by femoral nerve stimulation.

**Table 1:** Repeatability and reliability of parameters related to the contractile properties of unfatigued knee-extensor muscles

	Stimulation site	RC	CV	ICC_2,1_
Normalized peak relaxation rate	Motor cortex	1.8 s^−1^ (0.7–3.0)	5.6% (3.0–8.0)	0.933 (0.724–0.982)
	Femoral nerve	1.5 s^−1^ (0.7–2.2)	5.9% (2.6–9.2)	0.889 (0.603–0.968)
Time to peak relaxation	Motor cortex	9.5 ms (5.6–13.6)	3.3% (1.8–4.7)	0.891 (0.619–0.968)
Potentiated peak twitch amplitude	Femoral nerve	15.9 N (2.1–29.8)	3.9% (0.5–7.2)	0.828 (0.437–0.950)
Time to peak amplitude	Femoral nerve	3.2 ms (0.6–5.7)	1.3% (0.3–2.3)	0.932 (0.772–0.980)
Half-relaxation time	Femoral nerve	10.0 ms (2.2–17.7)	4.7% (1.4–8.0)	0.893 (0.645–0.969)

Values are means (95% confidence interval)

*RC* repeatability coefficient, *CV* coefficient of variation, *ICC*_*2,1*_ two-way random effects, absolute agreement intra-class correlation coefficients

### Force changes in fatigued knee-extensor muscles

**Fig. 3:**
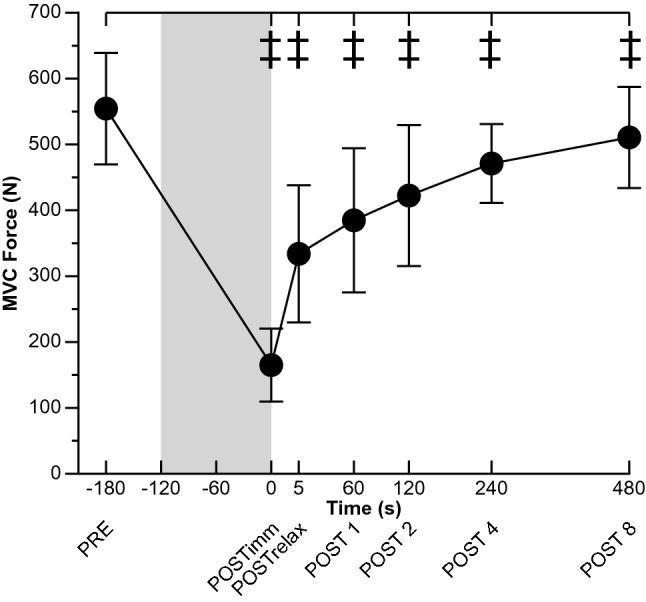
Changes in maximal voluntary contraction (MVC) force. The neuromuscular function evaluation was performed before (PRE) and at the end of the 2-min MVC. After the sustained contraction, a neuromuscular function evaluation was performed as an extension of the 2-min MVC (POSTimm) and additional evaluations were performed after 5 s of relaxation (POSTrelax) and 1 (POST 1), 2 (POST 2), 4 (POST 4), and 8 (POST 8) min after the end of the 2-min MVC. The shaded box indicates the sustained 2-min MVC and time ‘zero’ corresponds to the beginning of the recovery period. Values are means ± SD. For differences between time-points ^‡^*P* < 0.001

MVC force changes with fatigue are presented in Fig. 3. MVC force showed a time effect [*χ*^2^ (6) = 772.7, *P* < 0.001]. MVC force decreased from 554 ± 85 N at PRE to 165 ± 55 N at POSTimm (30 ± 10% of PRE values, *P* < 0.001), and remained lower than PRE throughout recovery (POST 8: 511 ± 77 N, 92 ± 7% of PRE values, *P* = 0.008).

### Resting twitch-derived parameters

Potentiated peak twitch amplitude showed a time effect [*χ*^2^ (6) = 935.8, *P* < 0.001]. The amplitude decreased from 144 ± 16 N at PRE to 40 ± 12 N at POST (28 ± 9% of PRE values, *P* < 0.001), and remained lower than PRE throughout recovery (POST 8: 109 ± 16 N, 76 ± 7% of PRE values, *P* < 0.001) (Table 2).

**Table 2:** Characteristics of the potentiated resting twitch evoked by femoral nerve stimulation before (PRE) and at the end of the 2-min MVC

Variable	PRE	POSTimm	POSTrelax	POST 1	POST 2	POST 4	POST 8
Potentiated peak twitch amplitude (N)	144 ± 16 (115/182)	40 ± 12^‡^ (17/67)	50 ± 17^‡^ (23/88)	84 ± 28^‡^ (44/139)	111 ± 27^‡^ (63/161)	120 ± 18^‡^ (94/143)	116 ± 19^‡^ (86/144)
Time to peak amplitude (ms)	88 ± 6 (79/99)	88 ± 5 (79/94)	92 ± 10 (79/94)	92 ± 6 (80/102)	90 ± 4 (79/97)	86 ± 5 (75/94)	81 ± 4 (72/87)
Half-relaxation time (ms)	72 ± 12 (58/93)	72 ± 13 (50/92)	72 ± 15 (51/101)	74 ± 13 (58/99)	74 ± 13 (56/101)	69 ± 11 (51/82)	65 ± 10 (51/87)
Normalized peak rate of relaxation (s^−1^)	− 9.4 ± 1.4 (− 7.1/− 11.3)	− 10.5 ± 1.7 (− 8.2/− 13.6)	− 10.1 ± 1.5 (− 7.4/− 12.3)	− 8.9 ± 1.3 (− 6.8/− 10.4)	− 8.9 ± 1.4 (− 6.8/− 11.2)	− 9.4 ± 1.6 (− 7.7/− 12.4)	− 11.2 ± 1.3 (− 9.3/− 13.8)

After the sustained contraction, a neuromuscular function evaluation was performed as an extension of the 2-min MVC (POSTimm) and additional evaluations were performed after 5 s of relaxation (POSTrelax) and 1 (POST 1), 2 (POST 2), 4 (POST 4), and 8 (POST 8) min after the end of the 2-min MVC. Values are means ± SD (min/max). For differences between time-points

^‡^*P* < 0.001

Time to peak amplitude of the potentiated peak twitch showed a time effect [*χ*^2^ (6) = 74.2, *P* < 0.001]. However, no time points were different than PRE (*P* ≥ 0.334) (Table 2).

Half-relaxation time of the potentiated peak twitch showed a time effect [*χ*^2^ (6) = 28.1, *P* < 0.001]. However, no time points were different than PRE (*P* ≥ 0.300) (Table 2).

Normalized peak relaxation rate showed a time effect [*χ*^2^ (1) = 49.3, *P* < 0.001]. However, no time points were different than PRE (all *P* = 1.000) (Table 2).

### TMS-derived parameters

For all participants at all time points, the duration of the silent period was sufficient to allow for measurement of the peak relaxation rate of muscle fibres (Table 3). Time to peak relaxation showed a time effect [*χ*^2^ (6) = 678.0, *P* < 0.001]. Time to peak relaxation increased from 107 ± 9 ms at PRE to 141 ± 33 ms at POSTimm (131 ± 28% of PRE values, *P* = 0.001), and recovered by POST 4 (110 ± 10 ms, 102 ± 7% of PRE values, *P* = 1.000).

**Table 3:** Comparison of the time to peak relaxation and the silent period evoked after delivery of the transcranial magnetic stimulation during maximal voluntary contractions

Variable	PRE	POSTimm	POSTrelax	POST 1	POST 2	POST 4	POST 8
Time to peak relaxation (ms)	107 ± 9 (90/120)	141 ± 33^†^ (60/179)	143 ± 15^‡^ (128/169)	129 ± 12^‡^ (111/148)	121 ± 11^‡^ (105/137)	110 ± 10 (97/128)	105 ± 7 (95/118)
*Rectus femoris* silent period (ms)	275 ± 58 (168/365)	313 ± 52^‡^ (221/398)	277 ± 64 (178/375)	267 ± 64 (159/356)	270 ± 64 (172/350)	275 ± 61 (182/365)	263 ± 64 (188/354)
*Vastus lateralis* silent period (ms)	277 ± 61 (166/364)	319 ± 53^‡^ (219/414)	277 ± 67 (147/375)	267 ± 62 (166/369)	273 ± 54 (192/363)	269 ± 65 (164/373)	266 ± 65 (183/375)

The neuromuscular evaluation was performed before (PRE) and at the end of the 2-min MVC. After the sustained contraction, a neuromuscular function evaluation was performed as an extension of the 2-min MVC (POSTimm) and additional evaluations were performed after 5 s of relaxation (POSTrelax) and 1 (POST 1), 2 (POST 2), 4 (POST 4), and 8 (POST 8) min after the end of the 2-min MVC. Values are means ± SD (min/max). For differences between time-points

^†^*P* < 0.01

^‡^*P* < 0.001

**Fig. 4:**
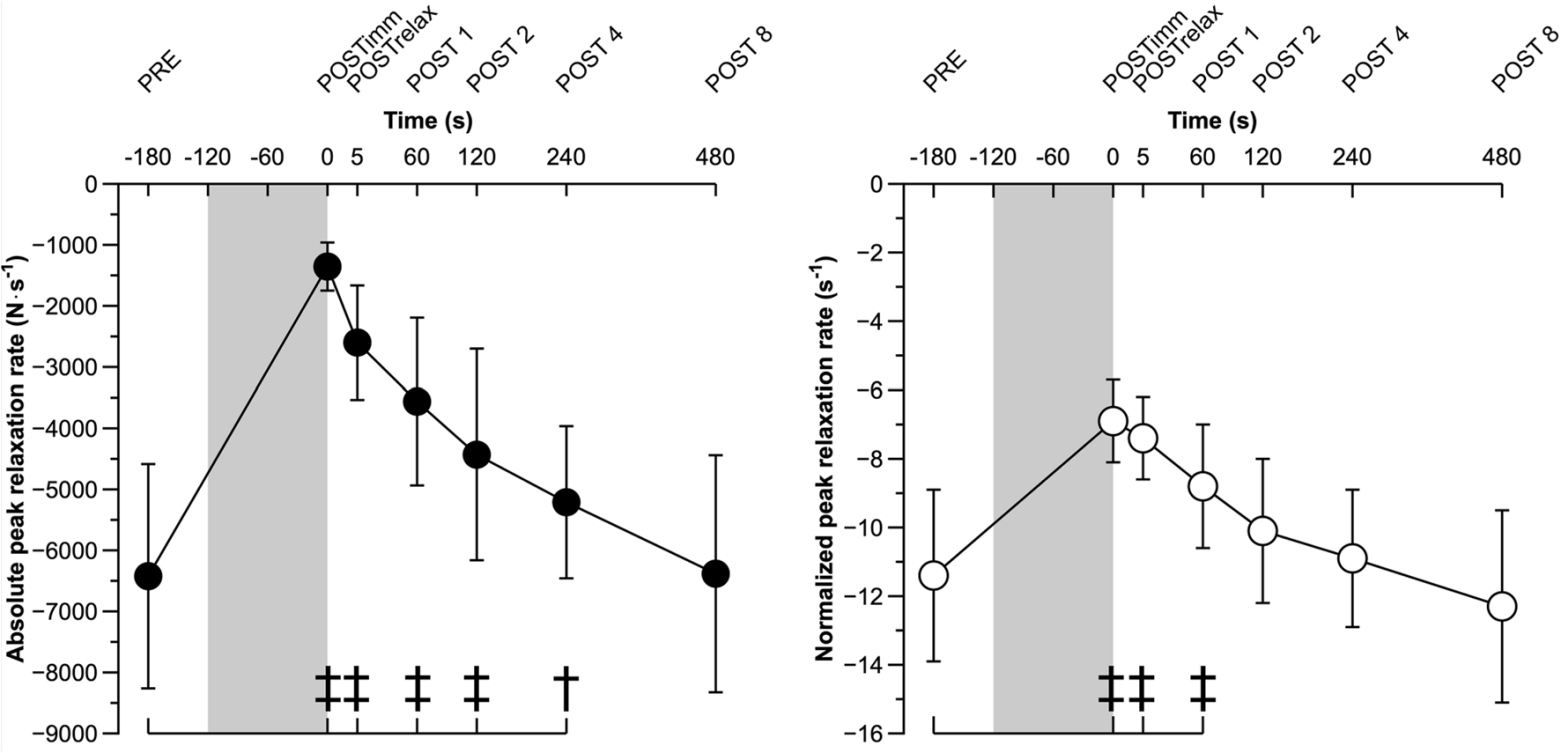
Changes in absolute and normalized peak relaxation rates (as determined from the TMS-induced decrease in force) during maximal voluntary contractions. The neuromuscular function evaluation was performed before (PRE) and at the end of the 2-min MVC. After the sustained contraction, a neuromuscular function evaluation was performed as an extension of the 2-min MVC (POSTimm) and additional evaluations were performed after 5 s of relaxation (POSTrelax) and 1 (POST 1), 2 (POST 2), 4 (POST 4), and 8 (POST 8) min after the end of the 2-min MVC. The shaded box indicates the sustained 2-min MVC and time ‘zero’ corresponds to the beginning of the recovery period. Values are means ± SD. For differences between time-points ^†^*P* < 0.01; ^‡^*P* < 0.001

Absolute and normalized peak relaxation rate changes with fatigue are presented in Fig. 4. The absolute peak relaxation rate showed a time effect [*χ*^2^ (6) = 565.0, *P* < 0.001]. Absolute peak relaxation rate decreased from − 6423 ± 1838 N·s^−1^ at PRE to − 1356 ± 394 N s^−1^ at POSTimm (22 ± 6% of PRE values, *P* < 0.001), and recovered by POST 8 (− 6383 ± 1943 N·s^−1^, 100 ± 15% of PRE values, *P* = 1.000).

The normalized peak relaxation rate showed a time effect [*χ*^2^ (6) = 89.1, *P* < 0.001]. Normalized peak relaxation decreased from − 11.5 ± 2.5 s^−1^ at PRE to − 6.9 ± 1.2 s^−1^ at POSTimm (63 ± 17% of PRE values, *P* < 0.001), and recovered by POST 2 (10.1 ± 2.0 s^−1^, 89 ± 13% of PRE values, *P* = 0.052).

## Discussion

The present study shows that the use of TMS delivered to the knee-extensor muscles can be used to measure muscle relaxation rates, both in unfatigued and fatigued knee extensors, an important muscle group for ambulatory and functional activities.

### Repeatability and reliability in unfatigued knee-extensor muscles

Our results show that repeatability of muscle relaxation rates determined from the decrease in force during the silent period following TMS delivery during MVC was similar to repeatability when compared to the falling phase of the resting twitch evoked by femoral nerve stimulation. This is shown by similar CV and RC in the muscle relaxation rates. The mean CV for the TMS-induced normalized peak relaxation rate is similar to that previously reported for finger flexors (Molenaar et al. 2018) and elbow flexors (Todd et al. 2007) in healthy participants. Furthermore, RCs were also similar to that previously reported for finger flexors (Molenaar et al. 2018) in healthy male participants. Relative reliability was also rated moderate to excellent, as indicated by a mean ICC_2,1_ value for TMS-induced peak relaxation rate of 0.933 (95% CI of 0.724–0.982). Similar results have previously been reported for finger flexors (Molenaar et al. 2018) in healthy male participants. Reliability refers to the amount of measurement error that is deemed acceptable for the effective use of a technique; and greater reliability implies that measurement differences are less likely to be due to measurement errors (Atkinson and Nevill 1998). In other words, greater reliability implies a greater sensitivity of the measurement in detecting true differences between participants.

### Peak relaxation rate in unfatigued knee extensors

The interruption of cortical output and motoneuron activity during the TMS-induced silent period implies that all KE muscle fibres that were previously contracting voluntarily (plus any additional muscle fibres recruited by TMS) were now relaxing. This relaxation rate reflects intrinsic contractile properties of KE rather than the ability of participants to withdraw neural drive as during voluntary relaxations (Todd et al. 2007). In other words, peak rate of muscle relaxation determined from the decrease in force during the silent period during voluntary contractions represents only intrinsic muscle relaxation properties. During complete relaxation of a muscle (i.e., during voluntary relaxation, the TMS-induced silent period or the relaxation phase following a twitch), the time course of relaxation is due to an interplay between the membrane-bound Ca^2+^ transport proteins and the sarcomeric proteins. This interplay presents a slow phase followed by a fast (almost mono-exponential) phase [for a comprehensive review see Poggesi et al. (2005)]. Previous studies reported faster mean relaxation rates to the present one in healthy young men for finger flexors [− 14.1 s^−1^ (Molenaar et al. 2018)], elbow flexors [− 13.5 s^−1^ (Hunter et al. 2006), − 12.9 s^−1^ (Hunter et al. 2008), − 14.3 s^−1^ (Molenaar et al. 2013)], and plantarflexors [− 13.1 s^−1^ (Yacyshyn et al. 2017)]. These faster relaxation rates could be due to a greater proportion of fast-twitch muscle fibres in the above-mentioned muscles compared to KE (Johnson et al. 1973).

### Peak relaxation rate in fatigued knee extensors

Absolute peak relaxation rates determined from the TMS-induced decrease in MVC force were affected by fatigue, slowing at the end of the 2-min MVC. After accounting for the participants’ force level, normalized peak relaxation rates showed similar results, declining by ~ 37% from PRE. With the use of TMS in KE, we showed fatigue-induced slowing of relaxation rate as previously reported during voluntary relaxation (e.g., Bigland-Ritchie et al. 1992), electrically induced relaxation (e.g., Bigland-Ritchie et al. 1983), and TMS-induced relaxation (e.g., Todd et al. 2005, 2007; Hunter et al. 2006, 2008; Molenaar et al. 2018). Since TMS-induced muscle relaxation rates only represent the intrinsic properties of a muscle, fatigue-induced changes in relaxation rate could have been due to a reduction in Ca^2+^ uptake by the sarcoplasmic reticulum (Gollnick et al. 1991). Indeed, muscle relaxation is initiated by a reduction in sarcoplasmic [Ca^2+^], and the efficiency of this process is dictated by three successive steps of Ca^2+^ removal: (1) dissociation of Ca^2+^ from troponin C, (2) translocation of Ca^2+^ to near the entry point of the sarcoplasmic reticulum, and (3) uptake of Ca^2+^ into the sarcoplasmic reticulum by the Ca^2+^ pump (Gordon et al. 2000). When fatigue reduced KE force by ~ 70%, we observed a decrease in the normalized peak relaxation rate. However, the normalized peak relaxation rate for the resting twitch evoked by femoral nerve stimulation did not show a fatigue-induced change (from − 9.4 ± 1.4 s^−1^ at PRE to − 10.5 ± 1.7 s^−1^ at POST, *P* = 1.000). Therefore, TMS-induced muscle relaxation rate reveals different results than the relaxation rate determined from the resting twitch evoked by femoral nerve stimulation in the fatigued KE, consistent with results previously observed for fatigued elbow flexors (Todd et al. 2007). Since muscle relaxation rate depends on the rate of detachment of cross-bridges during the relaxation process (Houston et al. 1987), in a fatigued state TMS-induced muscle relaxation rate may be more sensitive than the relaxation rate determined from the resting twitch evoked by femoral nerve stimulation to an altered muscle state.

### Limitations

Muscle relaxation properties can also be measured by high-frequency tetanic electrical stimulation, inducing a maximal sustained contraction (de Ruiter et al. 1999). However, this technique is very painful (especially in large muscle groups such as KE), making it unsuitable in clinical populations such as patients with neurological disorders. Recently, Molenaar et al. (2018) argued that voluntary relaxation after a finger-flexor MVC is a better representation of physiological muscle relaxation than electrical stimulation. This is because in voluntary motor unit recruitment, motor units are recruited according to the size principle (from small to large motor units) (Henneman 1957), whereas electrical stimulation recruits motor units in a nonselective, spatially fixed, and temporally synchronous pattern (from large to small motor units) (Gregory and Bickel 2005; Bergquist et al. 2011; Bickel et al. 2011). However, Molenaar et al. (2018) also compared TMS-induced muscle relaxation with voluntary muscle relaxation and TMS was more sensitive for assessing muscle relaxation rate.

## Conclusion

TMS provided suitable measures of peak relaxation rates in unfatigued KE. The use of TMS for measuring muscle relaxation during MVC also seems to be sufficiently sensitive and more appropriate than the resting twitch evoked by femoral nerve stimulation to reveal changes in KE contractile properties that one would expect after a sustained fatiguing isometric maximal contraction. Although resting twitches are deemed more practical than TMS-induced muscle relaxation rates (e.g., when the equipment is unavailable or participants have contraindications to the use of TMS), TMS may be useful to provide information about the properties of KE in its most functionally relevant state, that is during voluntary contraction (Todd et al. 2007). In other words, TMS-induced muscle relaxation rates reflect the same physiological mechanisms as the relaxation rate after a single electrical twitch but examine the muscle fibres when the central nervous system is driving voluntary muscle contraction. Furthermore, determination of the TMS-induced muscle relaxation rate allows tracking of fatigue-induced changes in intrinsic KE contractile properties without requiring the interruption of ongoing contractions that potentially can alter the intrinsic muscle contractile properties (Todd et al. 2005, 2007).

In conclusion, TMS-induced KE muscle relaxation is a reliable technique to measure intrinsic muscle relaxation properties. The quantification of TMS-induced KE muscle relaxation may help to inform research design and methodologies in TMS studies that directly investigate the muscle relaxation rate of KE, which is often implicated in exercise and human performance.
